# Long-term exposure to ambient particulate matter and its association with Alzheimer’s disease: influencing factors and a systematic review with meta-analysis

**DOI:** 10.3389/fpubh.2026.1757872

**Published:** 2026-02-04

**Authors:** Na Zhao, Zhenzhen Chen, Hong Sun

**Affiliations:** 1 Teaching and Research Section of Health Services and Management, School of Health and Elderly Care, Shandong Women’s University, Jinan, China; 2 Department of Emergency Medicine, Shandong Provincial Qianfoshan Hospital, Jinan, China; 3 Department of Rehabilitation, Yanji Traditional Chinese Medicine Hospital, Yanji, China

**Keywords:** air pollution, Alzheimer’s disease, long-term exposure, meta-analysis, particulate matter

## Abstract

**Background:**

Alzheimer’s disease (AD) poses a pressing public health burden globally. Evidence linking long-term ambient particulate matter exposure to AD risk remains inconsistent, necessitating systematic quantification to inform prevention policies.

**Methods:**

We searched PubMed, Embase, Web of Science, and Cochrane Library up to September 2025 for cohort studies with ≥1 year of particulate exposure (PM_2.5_, PM_10_, NO_2_, NO_x_, O_3_) and incident/diagnosed AD. Quality was assessed via the Newcastle–Ottawa Scale (NOS), with random-effects models pooling hazard ratios (HRs) and 95% CIs; subgroup analyses explored heterogeneity by study design, region, and follow-up duration.

**Results:**

Twenty-five high-quality (NOS ≥ 7/9) cohort studies involving over 170 million participants were included. Meta-analyses showed higher AD risk with each 5 μg/m^3^ increase in PM_2.5_ (HR = 1.24, 95%CI: 1.10–1.39), 10 μg/m^3^ in PM_10_ (HR = 1.16, 95%CI: 1.01–1.33), 10 μg/m^3^ in NO_2_ (HR = 1.06, 95%CI: 1.00–1.12), and 10 μg/m^3^ in NO_x_ (HR = 1.05, 95%CI: 1.03–1.07). For O_3_, four included studies showed no significant association (HR = 1.23, 95%CI: 0.65–2.31) with extremely high heterogeneity (*I*^2^ = 99.8%), indicating inadequate and unstable evidence. Subgroup analyses confirmed effect modification by study design, region, and follow-up duration; publication bias was low for most pollutants.

**Conclusion:**

We recommend high-risk population screening, stricter emission standards, and prioritizing emission reduction in AD primary prevention—aligning with global efforts to address environmental determinants of neurological health.

**Systematic review registration:**

PROSPERO with the registration number CRD420251174986, https://www.crd.york.ac.uk/prospero/display_record.php?ID=CRD420251174986.

## 1 Introduction

Alzheimer’s disease (AD) is the most prominent neurodegenerative disorder worldwide. Per the World Health Organization, global AD cases exceeded 55 million by 2019 and are projected to reach 78 million by 2030. Progressive cognitive decline, loss of daily living abilities, and surging care needs impose heavy family burdens and strain global medical resources and socioeconomic systems (1). AD’s pathological mechanism is complex—senile plaques from *β*-amyloid (Aβ) deposition, neurofibrillary tangles due to excessive tau phosphorylation, plus neuroinflammation and synaptic loss are core progression features, though its exact etiology remains incompletely elucidated (2, 3).

AD arises from the interplay of genetic and environmental factors. Particulate matter (PM) is a major environmental pollutant (4). Additionally, growing evidence links it to neurological damage and neurodegenerative diseases through multiple pathways (5). PM is classified by aerodynamic diameter: PM_10_ (≤10 μm), PM_2.5_ (≤2.5 μm), and ultrafine particles (PM_0.1_, ≤0.1 μm). PM_2.5_, due to its small size and large specific surface area, carries toxic pollutants (e.g., heavy metals, polycyclic aromatic hydrocarbons). These substances penetrate the alveolar and blood–brain barriers, acting directly on the central nervous system (6). Animal studies show PM_2.5_ induces lysosomal dysfunction, disrupts A*β* metabolism (e.g., PS1 upregulation), triggers neuroinflammation and myelin damage, and exacerbates AD pathology (7).

Despite numerous studies on long-term PM exposure and AD, evidence remains heterogeneous and controversial. Some prospective cohort studies report significant associations—for example, an Italian study of over 20,000 older adults found each 1 μg/m^3^ PM_10_ increase raised AD risk by 25% (HR = 1.25, 95% CI: 1.19–1.31) (8), and other research links PM_2.5_ to faster cognitive decline and altered AD biomarkers in older adults (9). However, cross-sectional or short-term studies often show no association. This may stem from differences in population baseline, exposure assessment, confounding control, or AD diagnostic criteria (10). Key gaps persist regarding PM’s impact on AD: (1) unclear AD risk differences from PM of varying sizes (e.g., PM_2.5_ vs. PM_10_); (2) scarce data from low- and middle-income countries (high pollution, large populations), limiting global evidence representativeness. To address these gaps, we conduct a systematic review and meta-analysis of global epidemiological studies on long-term ambient PM exposure and AD. Quantitative pooling of effect sizes will clarify their association strength, informing PM’s role in AD pathogenesis, targeted environmental interventions, and high-risk population screening.

## 2 Materials and methods

### 2.1 Study design and registration

This study is a systematic review and meta-analysis that synthesizes observational studies on the association between long-term ambient particulate matter exposure and AD incidence/diagnosis. It strictly adheres to the Preferred Reporting Items for Systematic Reviews and Meta-Analyses (PRISMA 2020 Statement). The study protocol was prospectively registered on PROSPERO (International Prospective Register of Systematic Reviews), with the registration number [CRD420251174986].

### 2.2 Literature search strategy

This study adopted a comprehensive search strategy using English databases to cover published studies worldwide on the association between long-term ambient particulate matter exposure and Alzheimer’s disease. Specifically, four core English databases were searched: PubMed, Embase, Cochrane Library, and Web of Science Core Collection, to ensure the comprehensiveness and accuracy of the search.

Search terms were constructed based on the dual themes of “exposure factor—outcome indicator” and adjusted to MeSH terms (e.g., for PubMed) or free-text terms according to the characteristics of each database, to maximize search comprehensiveness. Core search terms were categorized as follows: Particulate matter-related: “Particulate Matter,” “PM2.5,” “Ultrafine Particles,” “PM10,” “Particulate Air Pollutants”, “Airborne Particulate Matter”, “Ambient Particulate Matter”, “Ultrafine Fibers”; AD-related: “Alzheimer’s disease,” “AD,” “Familial Alzheimer Diseases”, “Focal Onset Alzheimer’s Diseases”, “Acute Confusional Senile Dementia”, “Senile Dementia.” Retrieval time frame: From the establishment of each database to September 12, 2025, to ensure the inclusion of the latest research. Language restriction: Only studies published in English. Key information (e.g., exposure assessment, outcome data, effect sizes) was co-translated by two researchers with sufficient language skills. This avoided language bias. Based on the PICO principle, the inclusion and exclusion criteria were clearly defined to ensure consistency in the definition of study participants, exposures, and outcomes. Details are presented in Table 1.

**Table 1:** Inclusion and exclusion criteria for literature.

Dimensions	Inclusion criteria	Exclusion criteria
Study population (P)	Adults aged ≥ 50 years (a high-risk population for AD); No diagnosis of AD or definite cognitive impairment at baseline; Clear population source (e.g., community-dwelling population, hospital-based population).	Individuals with a confirmed diagnosis of AD or other neurodegenerative diseases (e.g., Parkinson’s disease) at baseline; Children, adolescents, or individuals aged < 50 years; Special populations (e.g., occupationally exposed populations, to exclude non-environmental exposure).
Exposure factor (I)	Exposure type: Ambient particulate matter (PM_2.5_/PM_10_/etc.); Exposure duration: “Long-term exposure” defined as ≥ 1 year; Exposure assessment: Provision of clear exposure concentration data (e.g., annual average concentration, cumulative exposure); assessment methods include regional monitoring data, satellite retrieval models, and individual exposure monitoring.	Short-term exposure (exposure duration < 1 year, e.g., acute pollution events); Only mention “air pollution” without specifying the type or concentration of particulate matter; Unreliable exposure assessment methods (e.g., subjective reporting of exposure levels).
Outcome indicator (O)	Primary outcome: “Incidence” or “new diagnosis” of AD; Outcome diagnostic criteria: Adoption of internationally recognized criteria, such as the National Institute on Aging-Alzheimer’s Association (NIA-AA) criteria (48), World Health Organization (WHO) ICD-10/11 codes (G30.-), and the Diagnostic and Statistical Manual of Mental Disorders (DSM-IV/5).	Outcome: “Cognitive decline” or “Mild Cognitive Impairment (MCI)” (not confirmed AD); Without clear diagnostic criteria, or AD diagnosis based solely on scale scores (e.g., MMSE); Outcome: AD-related pathological indicators (e.g., *β*-amyloid (Aβ) deposition) rather than clinical incidence of AD.
Study type (S)	Cohort studies (prospective/retrospective cohorts) within observational studies, from which effect sizes and 95% confidence intervals (CIs) can be extracted.	Experimental studies (e.g., animal experiments, cell experiments) and review studies (systematic reviews, meta-analyses, commentaries); Cross-sectional studies (unable to determine the temporal relationship between exposure and outcome, which may confuse causality); Conference abstracts and abstract collections (incomplete data, making it impossible to extract effect sizes); Duplicate publications.

### 2.3 Literature screening and data extraction

Literature screening was independently done by two trained researchers (Na Zhao and Hong Sun), who have backgrounds in environmental epidemiology and neuroscience. Screening included two steps: ① Initial screening: Excluded studies that clearly failed inclusion criteria (e.g., animal experiments, cross-sectional studies) based on titles and abstracts. Potentially eligible studies were flagged for full-text review. ② Full-text screening: Obtained the flagged texts and assessed each against inclusion criteria individually.

Disagreements between the two on eligibility were resolved through discussion, or by third-party arbitration from a senior epidemiologist with relevant expertise if consensus failed to be reached, and a final list of included studies was finalized.

A self-designed Data Extraction Form was used, with extraction independently performed by the two and cross-checked for accuracy. Extracted information included: basic study details [first author, publication year, country/region, study type (prospective/retrospective cohort), sample size (total cohort)]; population characteristics [age (mean ± SD/median), gender ratio, AD diagnosis method, particulate type (PM_2.5_/PM_10_, etc.), exposure assessment method, average follow-up (years), exposure window, average exposure level]; and original effect sizes [hazard ratio (HR), relative risk (RR), odds ratio (OR)] and corresponding 95% CI.

### 2.4 Study quality assessment

Study quality was assessed using the Newcastle–Ottawa Scale (NOS) for observational studies—an internationally recognized tool to assess bias risk in cohort/case–control studies for systematic reviews, with eight items (total score: 9 points) that categorize studies as high quality (≥7 points), moderate quality (4–6 points), or low quality (≤3 points, excluded); its dimensions (for cohort studies) cover participant selection (4 points, 1 for defining exposure groups, 1 for representativeness of unexposed/exposed groups, 1 for no baseline AD, 1 for adequate sample size), comparability (2 points, 1 for adjusting key confounders, 1 for adjusting other potential ones like education and comorbidities), and outcome assessment (3 points, 1 for reliable outcome measurement such as clinical diagnosis + imaging confirmation, 1 for adequate follow-up ≥5 years, 1 for attrition <20% or proper handling). Two researchers (Na Zhao and Hong Sun) conducted the assessment independently, with disagreements resolved via discussion or third-party arbitration, and a final quality assessment table for included studies was generated.

### 2.5 Statistical analysis

Meta-analysis was performed using R software (version 4.4.2; R Foundation for Statistical Computing, Vienna, Austria) with the metafor package with two-tailed tests (*α* = 0.05). Heterogeneity was assessed via Q-test (*p* < 0.10 = significant heterogeneity) and *I*^2^ statistic: *I*^2^ < 25% (low): fixed-effects model; 25% ≤ *I*^2^ ≤ 50% (moderate): fixed-effects if study design/population consistent, else random-effects; *I*^2^ > 50% (high): random-effects (DerSimonian-Laird method), with subgroup analysis/meta-regression to explore sources. Random-effects models were preferred for synthesizing heterogeneous evidence as they account for both within-study sampling error and between-study variability, which is more robust for environmental epidemiology studies with inherent methodological differences.

For cohort studies, HR (95% CI) was used as effect size; multivariate-adjusted effect size was prioritized over unadjusted. For continuous exposures (e.g., PM_2.5_) with inconsistent dose increments (5/10 μg/m^3^), all effect sizes were first standardized to HR per fixed dose (e.g., 5 μg/m^3^ PM_2.5_) before random-effects pooling.

Heterogeneity sources were analyzed via meta-regression, subgroup, and sensitivity analyses. Meta-regression included exposure assessment method, follow-up duration, study region, and sample size as moderators to explore potential influencing factors. Subgroup analysis stratified by study region and methodology (with exposure assessment method as the core stratification factor), locating heterogeneity sources by re-pooling effect sizes and comparing heterogeneity indices. Sensitivity analysis excluded one study at a time to observe fluctuations in pooled HR (95% CI) and compared fixed- vs. random-effects results to verify stability.

Reliability of the results was verified by sensitivity analysis and publication bias assessment: Sensitivity analysis adopted the one-study removal approach, sequentially excluding each included study and re-pooling effect sizes to examine changes in hazard ratios (HRs) for result stability; publication bias was evaluated visually via funnel plots (*x*-axis = lnHR, *y*-axis = SE(lnHR), symmetry indicating no significant bias) and quantitatively using Egger’s test (*p* < 0.05 = significant bias), with the trim-and-fill method applied to adjust effect sizes if bias was detected.

## 3 Results

### 3.1 Literature screening results

This study retrieved a total of 1,428 relevant articles from databases: PubMed (*n* = 436), Embase (*n* = 518), Web of Science (*n* = 473), and Cochrane Library (*n* = 1). After removing duplicates using EndNote X9 software, 990 articles remained and proceeded to the initial screening stage.

During initial screening, 588 articles that did not meet the inclusion criteria were excluded based on titles and abstracts, with specific reasons including: animal experiments (*n* = 138), review articles (*n* = 158), cross-sectional studies (*n* = 76), outcomes limited to cognitive decline (*n* = 136), and short-term particulate matter exposure (<1 year, *n* = 80). A total of 402 articles were retained for full-text screening, during which 377 additional articles were excluded. The main exclusion reasons were: no clear AD diagnostic criteria (*n* = 149), failure to extract effect sizes and 95% confidence intervals (95% CIs, *n* = 52), study populations being occupationally exposed (*n* = 145), and conference abstracts with incomplete data (*n* = 31).

**Figure 1:**
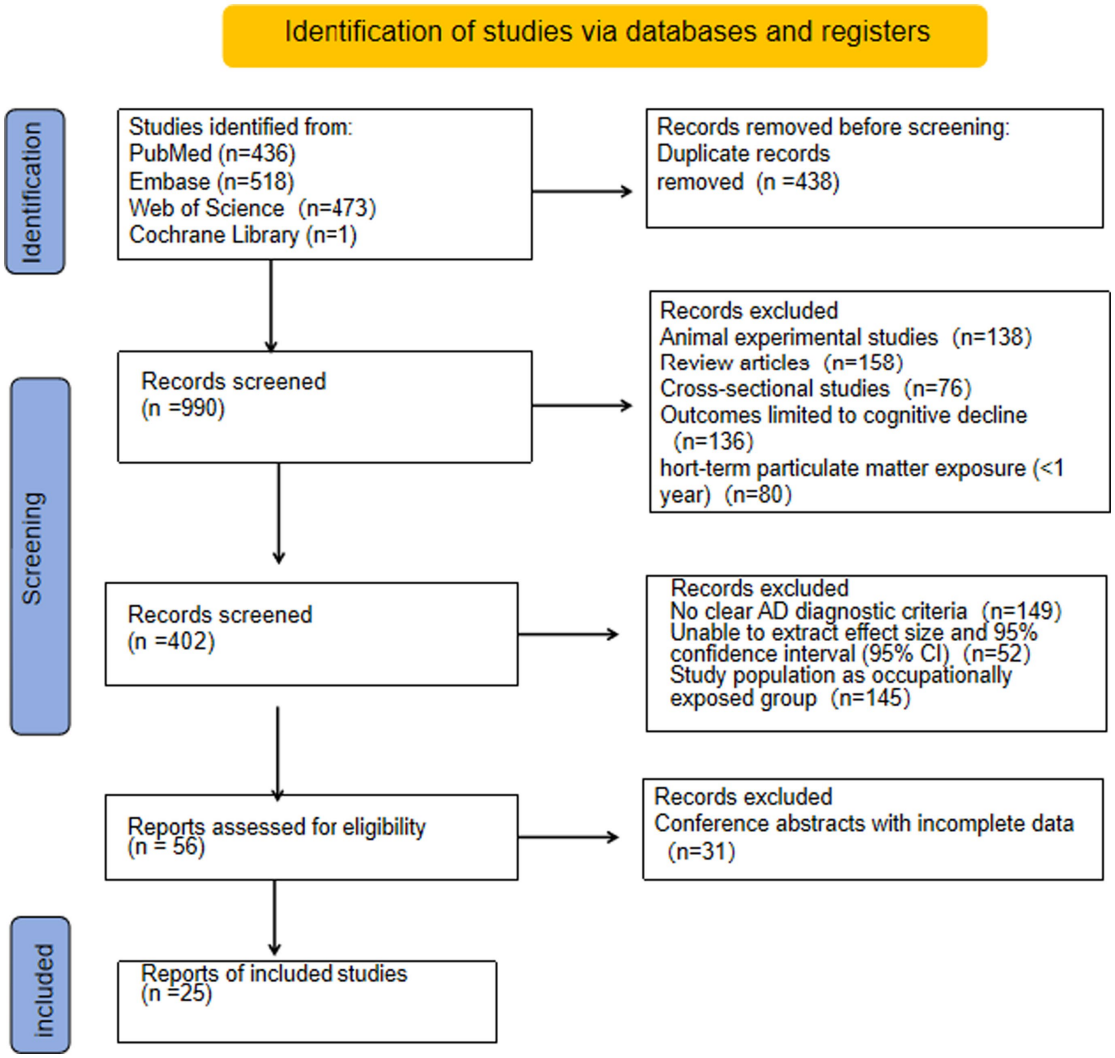
PRISMA flow diagram.

Finally, 25 studies that met the criteria were included. See Figure 1 for details of the literature screening process.

### 3.2 Basic characteristics of included studies

The 25 included studies (8, 10–33) were published between 2015 and 2025, including 15 prospective cohort studies (60.0%) and 10 retrospective cohort studies (40.0%). Geographically, 5 studies were from Asia (4 from China and 1 from South Korea), 19 from Europe [including 7 from the United Kingdom (UK)], and 1 from Oceania. Sample sizes ranged from 572 to 50,053,399 participants, with a total study population exceeding 171,896,112. Most participants were aged 50–85 years; some studies focused on single-gender populations (only males or only females), and gender ratios were reported in most cases. AD was defined via two approaches: diagnosis based on administrative databases and self-report. Regarding pollutant types, 22 studies analyzed PM_2.5_, 8 analyzed PM_10_, 12 analyzed NO_2_, 5 analyzed NO_x_, and only 4 analyzed O_3_. See Table 2 details.

**Table 2:** Summary of study characteristics.

First author (year)	Study design	State	Participants	Sex	Age (mean ± SD /median)	AD definition	Pollutants
Gialluisi et al. (2023) (8)	Prospective cohort study	Italy	24,195	12,695 (51.9%) female and 11,500 (48.1%) male	55.8 ± 12.0	Self-reported	PM_10_
Zhang et al. (2023) (10)	Prospective cohort study	United Kingdom (UK)	227,840	119,408 (52.4%) female and 108,432 (47.6%) male	60.1 ± 5.4	Administrative database	PM_2.5_, PM_2.5-10_, PM_10_, NO_2_, NO_x_
Jung et al. (2015) (11)	Prospective cohort study	Taiwan, China	95,690	44,119 (46.1%) female and 51,571 (53.9%) male	≥65	Self-reported	PM_2.5_, O_3_
Kioumourtzoglou et al. (2016) (12)	Retrospective cohort study	America	9,800,000	(57.3%) female and (42.7%) male	75.6 ± 7.6	Self-reported	PM_2.5_
Carey et al. (2018) (13)	Retrospective cohort study	UK	130,978	65,848 (50.3%) female and 65,130 (49.7%) male	50–79	Self-reported	PM_2.5_, NO_2_, O_3_
Oudin et al. (2019) (14)	Retrospective cohort study	Sweden	1,567	880 (56%) female and 687 (44%) male	Median: 69	Self-reported	NO_x_
Mortamais et al. (2021) (15)	Prospective cohort study	France	7,066	4,359 (61.7%) female and 2,707 (38.3%) male	Median: 73.4	Self-reported	PM_2.5_, NO_2_
Ran et al. (2021) (16)	Prospective cohort study	Hong Kong China	59,349	38,931 (65.6%) female and 20,418 (34.4%) male	≥65	Self-reported	PM_2.5_
Shi et al. (2021) (17)	Retrospective cohort study	America	24,689,818	14,557,997 (58.96%) female and 10,131,821 (41.04%) male	≥65	Self-reported	PM_2.5_, NO_2_, O_3_
Parra et al. (2022) (18)	Prospective cohort study	UK	187,194	98,459 (52.6%) female and 88,735 (47.4%) male	64.1 ± 2.84	Administrative database	PM_2.5_, NO_2_
Shi et al. (2022) (19)	Retrospective cohort study	America	19,200,000	40% female and 60% male	≥65	Self-reported	PM_2.5_
Trevenen et al. (2022) (20)	Prospective cohort study	Australia	11,243	100% male	72.1 ± 4.37	Self-reported	NO_2_, PM_2.5_
Yang et al. (2022) (21)	Prospective cohort study	China	1,545	806 (52.2%) female and 739 (47.8%) male	68.21 ± 4.81	Self-reported	PM_2.5_
Younan et al. (2022) (22)	Prospective cohort study	America	6,485	100% female	65–79	Self-reported	PM_2.5_
Chen et al. (2023) (23)	Prospective cohort study	UK	459,844	Not reported	50–69	Administrative database	PM_2.5_, PM_2.5-10_, PM_10_, NO_2_, NO_x_
Shim et al. (2023) (24)	Retrospective cohort study	Korea	1,36,361	766,909 (53.4%) female and 669,452 (46.6%) male	70.9 ± 4.9	Self-reported	PM_10_
Yuan et al. (2023) (25)	Prospective cohort study	UK	437,932	236,503 (54.3%) female and 199,341 (45.7%) male	Median: 58	Administrative database	PM_2.5_, PM_10_, NO_x_
Zhu et al. (2023) (26)	Prospective cohort study	China	29,025	17,180 (59.2%) female and 11,845 (40.8%) male	63.32 ± 9.41	Self-reported	PM_2.5_, PM_10_, NO_2_
Jutila et al. (2025) (27)	Prospective cohort study	UK	572	268 (47%) female and 304 (53%) male	Median: 70	Self-reported	PM_2.5_, NO_2_
Peters et al. (2024) (28)	Retrospective cohort study	Netherlands	10,735,734	5,507,858 (51.3%) female and 5,227,876 (48.7%) male	Mean: 54.3	Self-reported	PM_2.5_, PM_10_, NO_2_
Zhang et al. (2024) (29)	Prospective cohort study	UK	155,828	77,649 (50.01%) female and 78,179 (49.99%) male	64.09 ± 2.84	Self-reported	NO_2_, NO_x_, PM_2.5_, PM_10_, PM_2.5–10_
Qin et al. (2025) (30)	Retrospective cohort study	America	50,053,399	55.5% female and 44.5% male	≥65	Self-reported	M_2.5_, NO_2_, O_3_
Zhang et al. (2025) (31)	Retrospective cohort study	America	34,600,000	16,087,244 (57.9%) female and 11,676,349 (42.1%) male	≥65	Self-reported	PM_2.5_
Zheng et al. (2025) (32)	Prospective cohort study	UK	217,336	114,635 (52.7%) female and 102,701 (47.3%) male	64.1 ± 2.9	Administrativedatabase	PM_2.5_
Zhu et al. (2025) (33)	Retrospective cohort study	America	20,763,472	11,634,479 (56.0%) female and 9,128,993 (44.0%) male	76.60 ± 7.08	Self-reported	PM_2.5_

Details on exposure assessment and outcomes of the 25 included studies (8, 10–33) are shown in Table 3. The main exposure assessment method was land use regression (LUR) models (11 studies), followed by machine learning ensemble models (5 studies), direct monitoring (3 studies), atmospheric chemical transport models (1 study), and Bayesian maximum entropy spatiotemporal models (1 study). The average follow-up duration was 2–22.7 years, with most studies (22 studies) having a follow-up of 6–12 years. Exposure assessment windows included single-year, 5-year moving average, annual average during follow-up, 1-year average before baseline, and cumulative exposure. Effect sizes in all studies were presented as hazard ratios (HRs) and standardized for exposure increments in accordance with WHO guidelines.

**Table 3:** Exposure assessment and outcomes.

First author (year)	Exposure assessment methodology	Average follow-up years (mean ± SD/median)	Exposure assessment window	Average exposure level (mean ±SD/median)	Effect measure	Adjusted
Gialluisi et al. (2023) (8)	Kriging model	Mean: 11.17	Average concentration during the 2006–2018 follow-up period	PM_10_ = 11.6 μg/m^3^	HR	PM_10_ = 1.25 (1.19–1.31)
Zhang et al. (2023) (10)	Land use regression model	8.9 years (2006–2015)	Baseline year average pollutant concentration	PM_2.5_ = 9.9 ± 1.0 μg/m^3^; PM_10_ = 19.1 ± 1.9 μg/m^3^; NO_2_ = 28.2 ± =8.8 μg/m^3^	HR	PM_2.5_ = 1.20 (0.88–1.65) PM_10_ = 1.41 (0.98–2.03) NO_2_ = 1.16 (1.07–1.26) NO_x_ = 1.04 (0.99–1.08)
Jung et al. (2015) (11)	Direct monitoring (ground)	10 years (2001–2010)	2000–2010 average concentration	O_3_ = 88.97 ppb (IQR: 10.91); PM_2.5_ = 34.40 μg/m^3^ (IQR: 4.34)	HR	PM_2.5_ = 2.72 (2.09–2.39) O_3_ = 3.12 (2.92–3.33)
Kioumourtzoglou et al. (2016) (12)	Direct monitoring (ground)	11 years (1999–2010)	Annual dynamic concentration	PM_2.5_ = 12.0 ± 1.6 μg/m^3^	HR	PM_2.5_ = 2.00 (1.70–2.35)
Carey et al. (2018) (13)	KCLurban dispersion model	6.9 years (2005–2013)	Single year (2004)	NO_2_ = 37.1 μg/m^3^ (IQR: 5.7); PM_2.5=_15.7 μg/m^3^ (IQR: 0.8); O_3_ = 38.0 μg/m^3^ (IQR: 3.9)	HR	PM_2.5_ = 1.70 (1.32–2.55) NO_2_ = 1.32 (1.08–1.60) PM_2.5_ = 1.70 (1.32–2.55) O_3_ = 0.78 (0.66–0.92)
Oudin et al. (2019) (14)	Land use regression model	15 years (1993–1995 to 2008–2010)	1993–1995 average concentration	NO_x_ = 17 μg/m^3^	HR	NO_x_ = 1.41 (1.01–1.98)
Mortamais et al. (2021) (15)	Land use regression model	Median: 10.0 years; Longest: 12 years	Past 10-Year moving average concentration	PM_2.5_ = 21.9 ± 2.6 μg/m^3^ (14.6–31.3); NO_2_ = 34.2 ± 7.5 μg/m^3^ (12.8–91.8)	HR	PM_2.5_ = 1.20 (1.09–1.32) NO_2_ = 1.02 (0.94–1.12)
Ran et al. (2021) (16)	Satellite-based spatiotemporal model	Median: 10.4 years (1998–2001 to 2011)	1998–2001 average concentration	PM_2.5_ = 34.36 μg/m^3^ (IQR: 3.8)	HR	PM_2.5_ = 1.04 (0.93–1.17)
Shi et al. (2021) (17)	Machine learning ensemble model	Median: 7 years (2000–2018)	5-year before diagnosis moving average concentration	PM_2.5_ = 9.3 ± 3.2 μg/m^3^; NO_2_ = 17.1 ± 11.6 ppb; O_3_ = 42.6 ± 5.3 ppb	HR	PM_2.5_ = 1.12 (1.11–1.14) NO_2_ = 1.03 (1.02–1.04) O_3_ = 0.81 (0.80–0.82)
Parra et al. (2022) (18)	Land use regression model	7.04 ± 2.84	Single year (2010)	PM_2.5_ = 9.86 μg/m^3^ (IQR: 1.25); NO_2_ = 25.45 μg/m^3^ (IQR: 9.47)	HR	PM_2.5_ = 1.87 (1.27–2.78) NO_2_ = 1.13 (1.03–1.75)
Shi et al. (2022) (19)	Machine learning ensemble model	17 years (2000–2017)	Annual average concentration	PM_2.5_ = 9.58 μg/m^3^	HR	PM_2.5_ = 1.15 (1.14–1.16)
Trevenen et al. (2022) (20)	Land use regression model	Longest: 22.7 years	Baseline year average concentration and annual moving average concentration	NO_2_ = 13.5 ± 4.41 μg/m^3^; PM_2.5_ = 4.54 ± 1.56 μg/m^3^	HR	PM_2.5_ = 0.94 (0.78–1.12) NO_2_ = 0.93 (0.82–1.04)
Yang et al. (2022) (21)	Machine learning ensemble model	2 years (2018–2020)	Average concentration (2013–2017)	PM_2.5_ = 35.73 ± 2.95 μg/m^3^	HR	PM_2.5_ = 1.01 (0.99–1.04)
Younan et al. (2022) (22)	Bayesian maximum entropy spatiotemporal model	8.3 ± 3.5	1999–2010 annual dynamic exposure1	PM_2.5_: IQR = 3.73 μg/m^3^	HR	PM_2.5_ = 1.39 (1.03–1.89)
Chen et al. (2023) (23)	Land use regression model	Median: 11.7 years (Longest: up to March 2021)	2006–2010 average concentration	PM_2.5_ = 8.8 (8.4–9.2) μg/m^3^; PM_10_ = 17.3 (16.5–18.1) μg/m^3^; NO_2_ = 19.7 (17.0–22.0) μg/m^3^; NO_x_ = 28.4 (23.9–32.9) μg/m^3^	HR	PM_2.5_ = 1.12 (1.07–1.17) PM_10_ = 1.40 (1.13–1.74) NO_2_ = 1.13 (1.08–1.18) NO_x_ = 1.05 (1.03–1.08)
Shim et al. (2023) (24)	Direct monitoring (ground)	8.6 ± 4.1	Follow-up period annual average concentration	PM_10_ = 48.4 ± 7.7 μg/m^3^	HR	PM_10_ = 0.99 (0.98–1.00)
Yuan et al. (2023) (25)	Land use regression model	Median: 12.01 years (longest: up to March 2021)	Single year (2010)	PM_2.5_ = 9.9 μg/m^3^ (9.3–10.6); PM_10_ = 16 μg/m^3^ (15.2–17); NO_x_ = 42.1 μg/m^3^ (34.1–50.6)	HR	PM_2.5_ = 1.13 (0.97–1.31) PM_10_ = 1.79 (0.21–11.21) NO_x_ = 1.26 (1.08–1.48)
Zhu et al. (2023) (26)	Land use regression model	Median: 5.82 years	1-Year before baseline average concentration	PM_2.5_ = 34.55 μg/m^3^ (IQR 5.32); PM_10_ = 52.76 μg/m^3^ (IQR: 7.44); NO_2_ = 25.57 μg/m^3^ (IQR: 11.09)	HR	PM_2.5_ = 1.38 (1.08–1.77) PM_10_ = 1.22 (0.99–1.51) NO_2_ = 1.08 (0.76–1.55)
Jutila et al. (2025) (27)	Atmospheric chemical transport model	Median: 11.26 years	Specific time points (1935, 1950, 1970) + cumulative exposure (1935–1950, 1935–1970, etc.)	PM_2.5_ = 3.98 μg/m^3^	HR	PM_2.5_ = 0.98 (0.71–1.37) NO_2_ = 1.73 (0.84–3.56)
Peters et al. (2024) (28)	Land use regression model and dispersion model	6.6 years (71 million person—years, 2013–2019)	Single year (2016)	PM_2.5_ = 1.47 μg/m^3^; NO_2_ = 6.52 μg/m^3^	HR	PM_2.5_ = 0.87 (0.81–0.93) PM_10_ = 0.91 (0.80–1.03) NO_2_ = 0.93 (0.90–0.98)
Zhang et al. (2024)(29)	Land use regression model	12 years (2006–2021)	Single year (2010)	NO_2_ = 25.42 μg/m^3^; NO_x_ = 41.09 μg/m^3^; PM_2.5_ = 9.86 μg/m^3^; PM_10_ = 15.18 μg/m^3^	HR	PM_2.5_ = 1.46 (1.20–1.78) PM_10_ = 1.36 (0.78–2.37) NO_2_ = 1.09 (1.04–1.16) NO_x_ = 1.05 (1.02–1.08)
Qin et al. (2025) (30)	Machine learning ensemble model	8.3 years (2000–2016)	Follow-up period annual average concentration	PM_2.5_ = 11.6 μg/m^3^; NO_2_ = 22.7 ppb; O_3_ = 46.9 ppb	HR	PM_2.5_ = 1.13 (1.10–1.17) NO_2_ = 1.02 (1.00–1.03) O_3_ = 1.14 (1.03–1.25)
Zhang et al. (2025) (31)	Machine learning ensemble model	18 years (2000–2018)	5-year moving average concentration	PM_2.5_ = 9.87 μg/m^3^	HR	PM_2.5_ = 1.09 (1.08–1.01)
Zheng et al. (2025) (32)	EMEP4UK model	Median: 12.1 years	Follow-up period annual average concentration	PM_2.5_ = 9.06 μg/m^3^	HR	PM_2.5_ = 1.1 (1.02–1.19)
Zhu et al. (2025) (33)	Land use regression model	Median: 3 years (2018–2020)	Single year (2017)	PM_2.5_ = 7.1 μg/m^3^	HR	PM_2.5_ = 1.10 (1.08–1.12)

Effect sizes were expressed as estimates from the most adjusted model, and standardized for exposure concentration increments in accordance with the World Health Organization (WHO) Annual Air Quality Guidelines: 5 μg/m^3^ increase in PM_2.5_, 10 μg/m^3^ increase in PM_10_, 10 μg/m^3^ increase in nitrogen dioxide, 10 μg/m^3^ increase in nitrogen oxides, and 60 μg/m^3^ increase in ozone. HR, hazard ratio; NO_2_, nitrogen dioxide; NO_X_, nitrogen oxides; O_3_, ozone; PM, particulate matter; ppb, parts per billion; SD, standard deviation; IQR, interquartile range.

### 3.3 Quality assessment results of included studies

Bias risk assessment of the 25 included studies (8, 10–33) was performed using the NOS for cohort studies, covering three core dimensions: participant selection, inter-group comparability, and outcome assessment. The re-evaluated scores showed a reasonable distribution across high-quality tiers: 14 studies achieved a full score of 9/9 (fully meeting all NOS criteria), 8 studies scored 8/9 (with minor deductions for limited adjustment of secondary confounders or unclear follow-up attrition rates), and 3 studies scored 7/9 (with deductions for specific population selection and partial confounding adjustment). All included studies attained a NOS score ≥7/9, indicating high-quality evidence that guarantees the reliability of the meta-analysis results (Table 4).

**Table 4:** Newcastle–Ottawa scale quality assessment.

First author (year)	Selection (*n*/4)	Comparability (*n*/2)	Outcome (*n*/3)	Total (*n*/9)	Quality grade
Gialluisi et al. (2023) (8)	4	2	3	9	High
Zhang et al. (2023) (10)	4	2	3	9	High
Jung et al. (2015) (11)	4	1	3	8	High
Kioumourtzoglou et al. (2016) (12)	4	2	3	9	High
Carey et al. (2018) (13)	4	2	2	8	High
Oudin et al. (2019)(14)	3	2	3	8	High
Mortamais et al. (2021) (15)	4	2	3	9	High
Ran et al. (2021) (16)	4	1	3	8	High
Shi et al. (2021) (17)	4	2	3	9	High
Parra et al. (2022) (18)	4	2	3	9	High
Shi et al. (2022) (19)	4	2	3	9	High
Trevenen et al. (2022) (20)	3	1	3	7	High
Yang et al. (2022) (21)	4	1	3	8	High
Younan et al. (2022) (22)	3	1	3	7	High
Chen et al. (2023) (23)	4	2	3	9	High
Shim et al. (2023) (24)	4	2	2	8	High
Yuan et al. (2023) (25)	4	2	3	9	High
Zhu et al. (2023) (26)	4	1	3	8	High
Jutila et al. (2025) (27)	4	2	2	8	High
Peters et al. (2024) (28)	4	2	3	9	High
Zhang et al. (2024) (29)	4	2	3	9	High
Qin et al. (2025) (30)	4	2	3	9	High
Zhang et al. (2025) (31)	4	1	3	8	High
Zheng et al. (2025) (32)	4	2	3	9	High
Zhu et al. (2025) (33)	4	2	2	8	High

### 3.4 Relationship between 5 μg/m^3^ increase in PM_2.5_ and incident AD risk

We used a random-effects model because heterogeneity across studies was extremely high (*I*^2^ = 97.8%, *p* < 0.001). This heterogeneity violates the assumption of a common true effect needed for fixed-effects models. The random-effects model accounts for both within-study sampling error and between-study variability, making results more reliable and generalizable. For meta-analysis of 22 studies (10–13, 15–23, 25–33) to assess the association between a 5 μg/m^3^ increase in PM_2.5_ exposure and incident AD risk, results showed a significant positive association: pooled HR = 1.24 (95% CI = 1.10–1.39, *p* < 0.001).

We used meta-regression to explore heterogeneity, with moderators including exposure assessment method, follow-up duration, study region, and sample size. The overall model was significant (QM = 101.26, *p* < 0.0001), explaining 86.05% of heterogeneity (*R*^2^ = 86.05%). Though no single moderator reached significance, direct ground monitoring (DirectMon, *p* = 0.0986) and Bayesian maximum entropy spatiotemporal model (*p* = 0.0868) showed marginal associations (positive estimates); other factors (follow-up, region, sample size) had no significant contributions. Residual *I*^2^ remained high (97.36%), but tau^2^ decreased from 0.0693 to 0.0097, confirming moderators as major heterogeneity sources.

**Figure 2:**
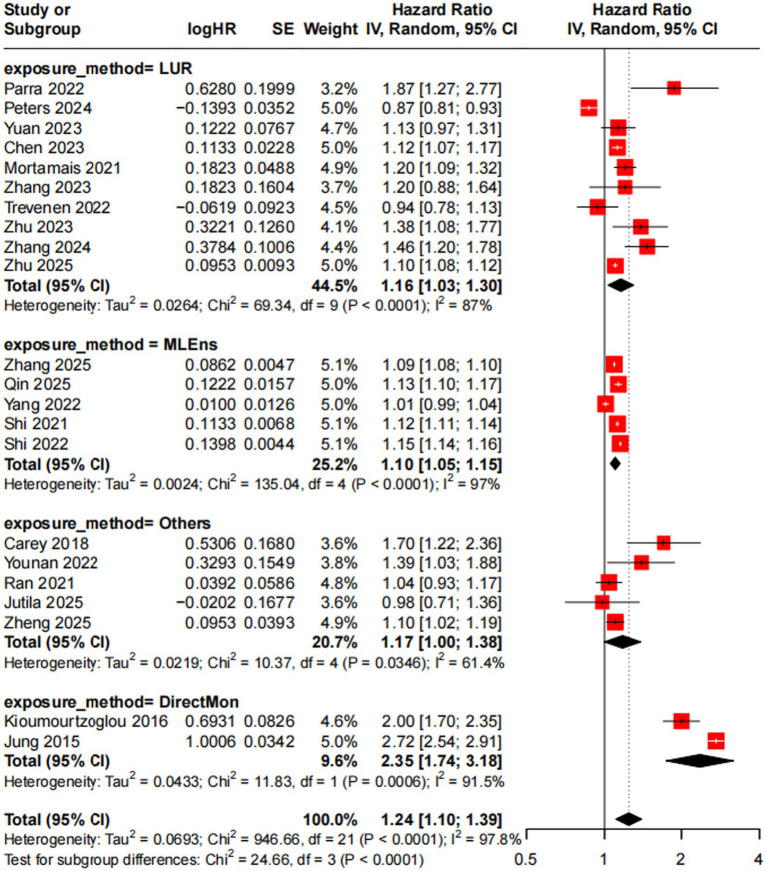
Forest plot of meta-analysis on the association between a 5 μg/m^3^ increase in PM_2.5_ and incident AD risk. LUR, land-use regression model; MLEns, machine learning ensemble model; DirectMon, direct monitoring ground; “Others” subgroup includes rare exposure methods (KCLurban dispersion model, Bayesian maximum entropy spatiotemporal model, satellite-based spatiotemporal model, atmospheric chemical transport model, EMEP4UK model).

Subgroup analysis (by simplified exposure methods: LUR, MLEns, DirectMon, Others) further verified significant between-subgroup differences (Q = 24.66, *p* < 0.0001): DirectMon (*n* = 2) had the strongest association (HR = 2.35, 95% CI: 1.74–3.18), followed by LUR (k = 10, HR = 1.15) and MLEns (*n* = 5, HR = 1.10); the “Others” subgroup (*n* = 5) showed no significant association (HR = 1.17, 95% CI: 0.996–1.38) due to rare methods with small samples. These subgroup trends aligned with meta-regression, confirming exposure assessment method as the core heterogeneity source (Figure 2).

### 3.5 Relationship between a 10 μg/m^3^ increase in PM_10_ and incident AD risk

**Figure 3:**
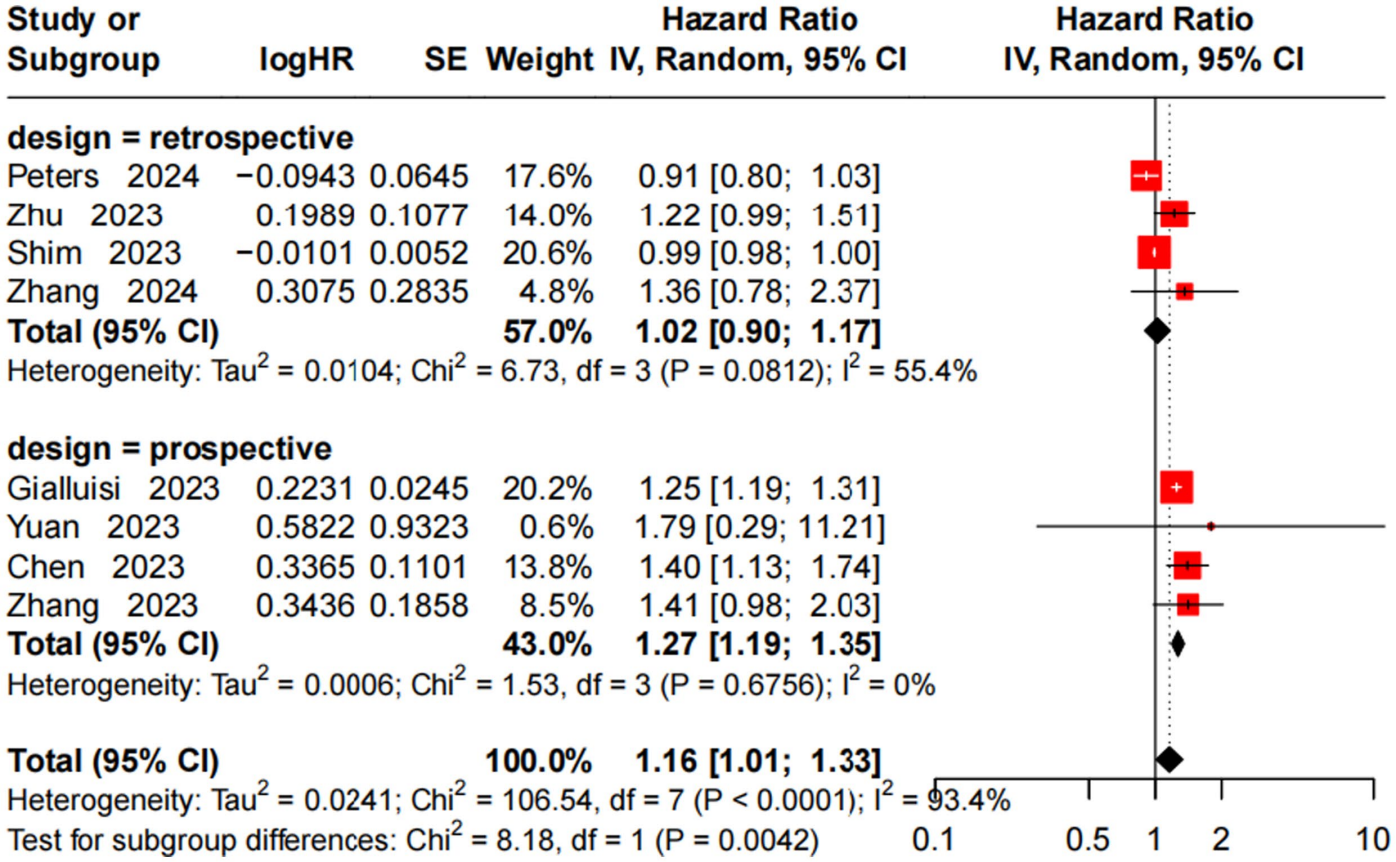
Forest plot of meta-analysis on the association between a 10 μg/m^3^ increase in PM_10_ and incident AD risk.

A random-effects model was adopted given the high between-study heterogeneity (*I*^2^ = 93.4%, *p* < 0.0001), which accounts for both sampling error and inherent variability across studies. This model was used to analyze the association between a 10 μg/m^3^ increase in PM10 exposure and incident AD risk, with subgroup stratification by study design (retrospective vs. prospective). Retrospective subgroup (4 studies) (10, 24, 26, 28): no significant association (HR = 1.02, 95% CI = 0.90–1.17) with moderate heterogeneity (*I*^2^ = 55.4%, *p* = 0.081). Prospective subgroup (4 studies) (8, 10, 23, 25): significant positive association (pooled HR = 1.27, 95% CI = 1.19–1.35) with very low heterogeneity (*I*^2^ = 0%, *p* = 0.676). Overall (8 studies): significant association (HR = 1.16, 95% CI = 1.01–1.33) with high heterogeneity (*I*^2^ = 93.4%, *p* < 0.0001). Subgroup heterogeneity test (*p* = 0.004) identified study design as an important heterogeneity source, with a more pronounced PM10-AD association in prospective studies (Figure 3).

### 3.6 Relationship between a 10 μg/m^3^ increase in NO_2_ and incident AD risk

**Figure 4:**
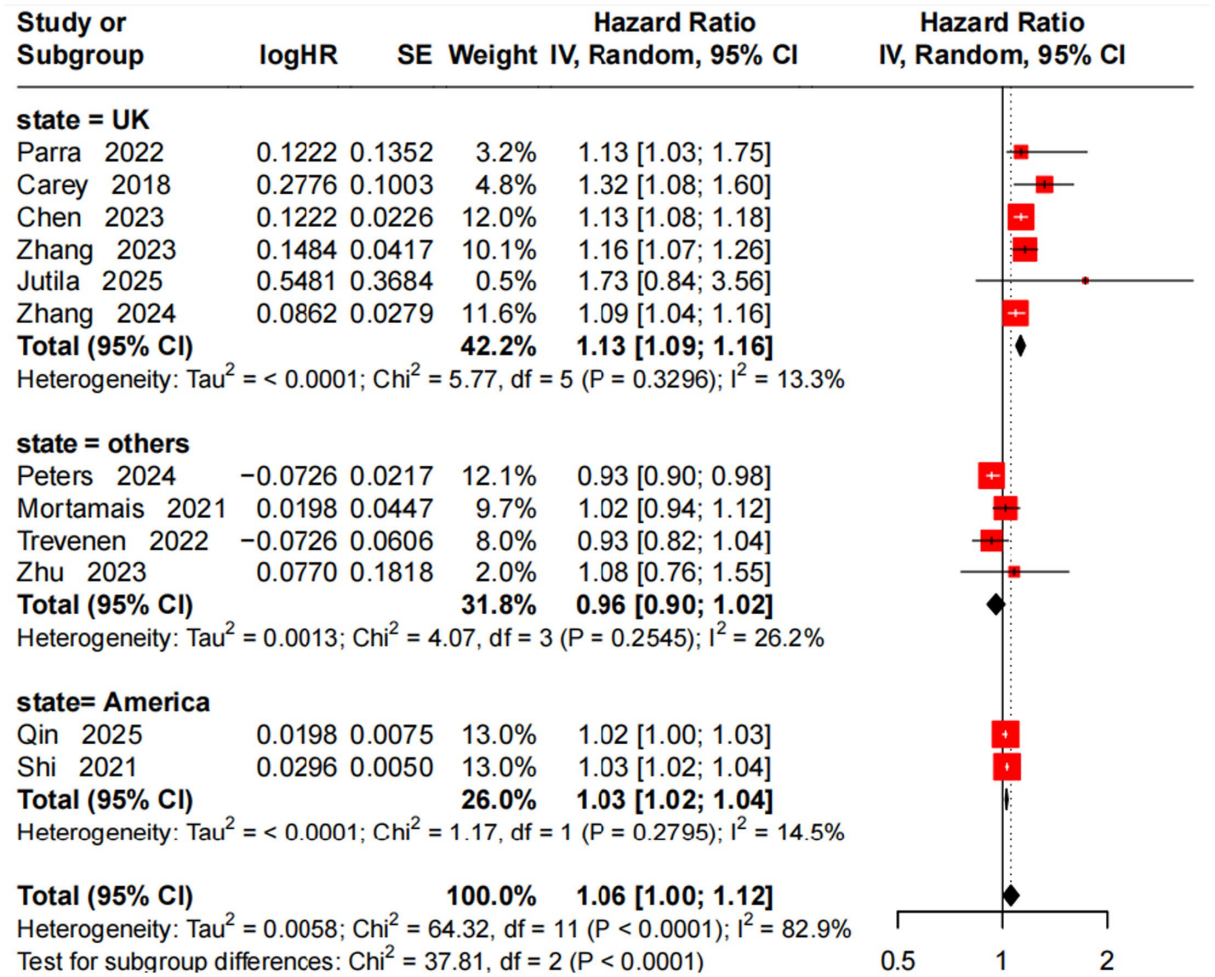
Forest plot of meta-analysis on the association between a 10 μg/m^3^ increase in NO_2_ and incident AD risk.

A random-effects model analyzed the association between a 10 μg/m^3^ NO_2_ increase and incident AD risk, with subgroup stratification by region. UK subgroup (6 studies) (10, 13, 18, 23, 27, 29): significant association (HR = 1.13, 95% CI = 1.09–1.16), low heterogeneity (*I*^2^ = 13.3%, *p* = 0.33). Others subgroup (4 studies) (15, 20, 26, 28): no significant association (HR = 0.96, 95% CI = 0.90–1.02), moderate heterogeneity (*I*^2^ = 26.2%, *p* = 0.25). America subgroup (2 studies) (17, 30): slight positive association (HR = 1.03, 95% CI = 1.02–1.04), low heterogeneity (*I*^2^ = 14.5%, *p* = 0.280). Overall (12 studies): significant association (HR = 1.06, 95% CI = 1.00–1.12), high heterogeneity (*I*^2^ = 82.9%, *p* < 0.001). Subgroup difference test (*p* < 0.0001) identified region as an important heterogeneity source, with a more significant NO2-AD association in the UK (Figure 4).

### 3.7 Relationship between a 10 μg/m^3^ increase in NO_x_ and incident AD risk

**Figure 5:**
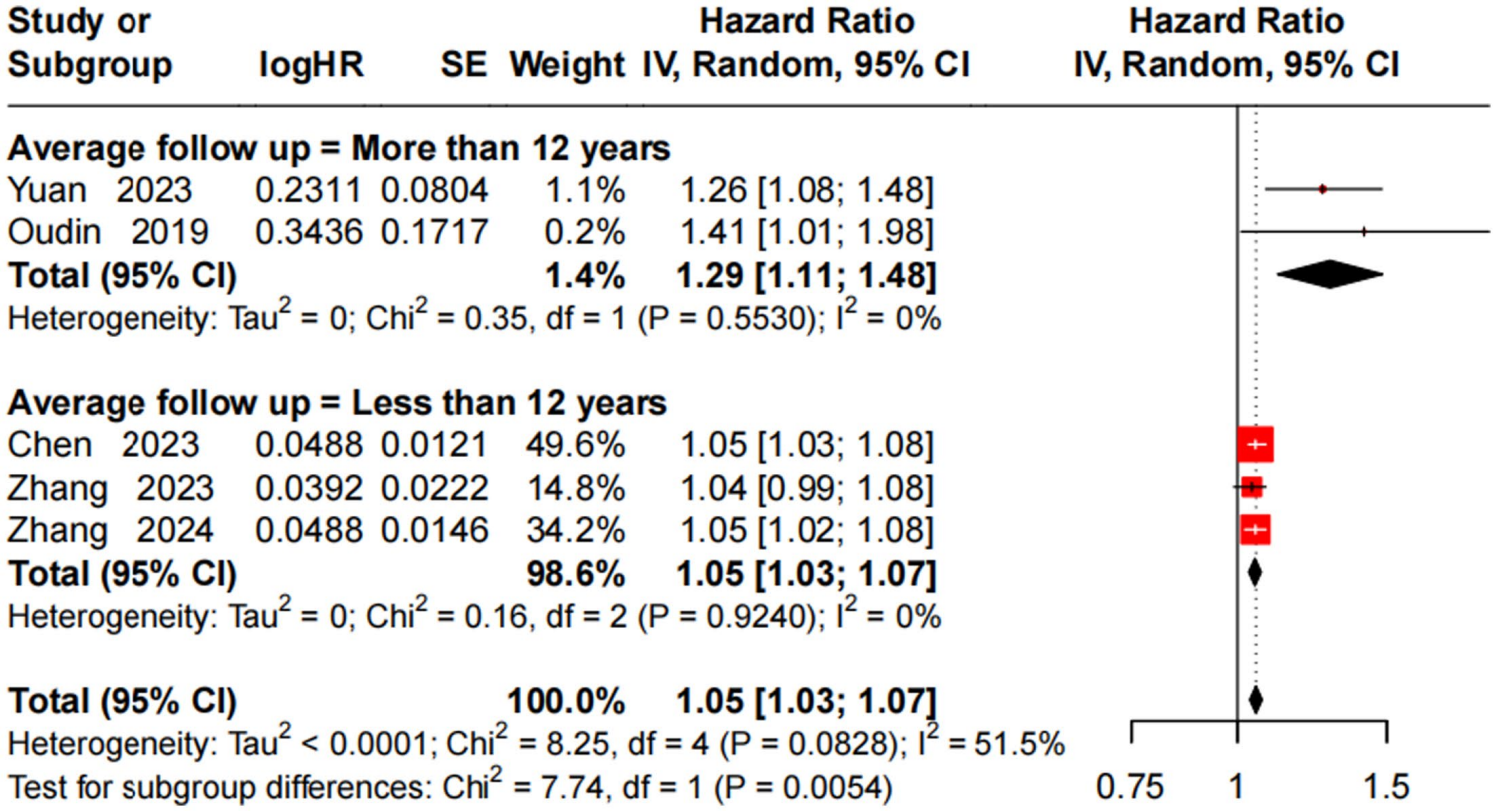
Forest plot of meta-analysis on the association between a 10 μg/m^3^ increase in NO_x_ and incident AD risk.

A random-effects model analyzed the association between a 10 μg/m^3^ NOₓ increase and incident AD risk, with subgroup stratification by average follow-up duration. Follow-up >12 years (2 studies) (14, 25): significant association (HR = 1.29, 95% CI = 1.11–1.48), no heterogeneity (*I*^2^ = 0%, *p* = 0.553). Follow-up <12 years (3 studies) (10, 23, 29): significant association (HR = 1.05, 95% CI = 1.03–1.07), no heterogeneity (*I*^2^ = 0%, *p* = 0.924). Overall (5 studies): significant association (HR = 1.05, 95% CI = 1.03–1.07), moderate heterogeneity (*I*^2^ = 51.5%, *p* = 0.083). Subgroup difference test (*p* = 0.005) identified follow-up duration as an important heterogeneity source, with a stronger NOₓ-AD association in the long follow-up subgroup (>12 years) (Figure 5).

### 3.8 Relationship between a 60 μg/m^3^ increase in O_3_ and incident AD risk

**Figure 6:**
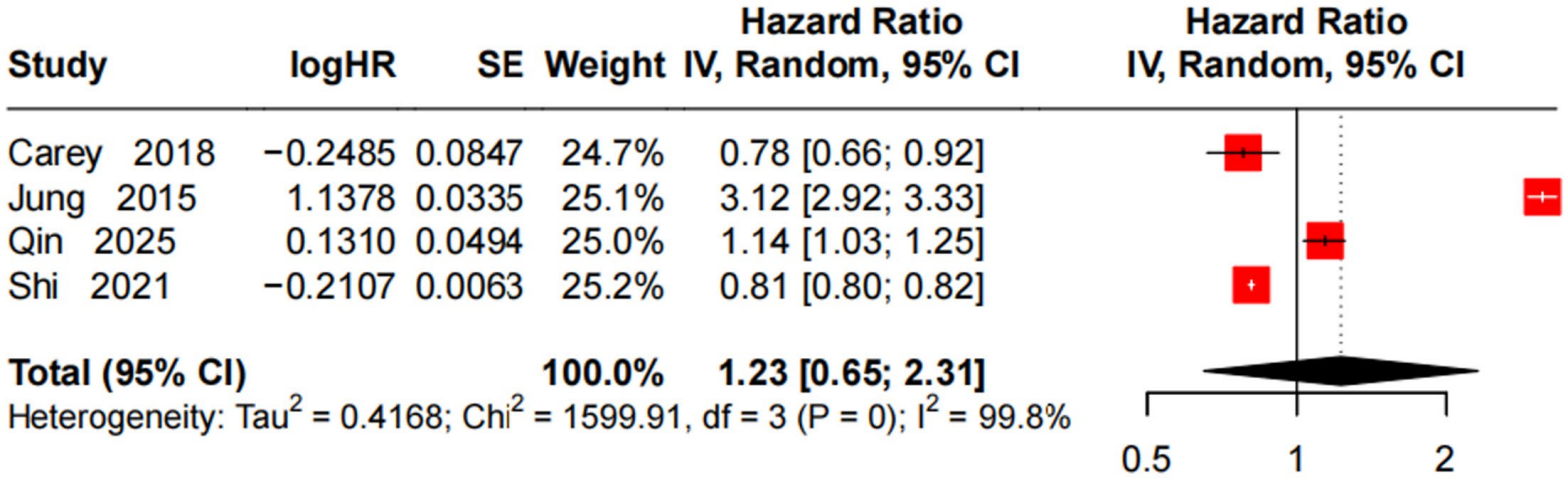
Forest plot of meta-analysis on the association between a 60 μg/m^3^ increase in O_3_ and incident AD risk.

A random-effects model analyzed 4 studies (11, 13, 17, 30) to assess the association between a 60 μg/m^3^ O3 increase and incident AD risk. Results showed no statistically significant association (HR = 1.23, 95% CI = 0.65–2.31, *p* > 0.05), with extremely high heterogeneity (*I*^2^ = 99.8%, *p* < 0.001). Notably, the included studies exhibited opposing effect directions: one study reported a strong positive association (HR = 3.12), while another showed a non-significant negative association (HR = 0.78). Given the small number of included studies (*n* = 4), conflicting effect directions, and extreme heterogeneity, the current evidence is inadequate and unstable to confirm or refute an association between long-term O_3_ exposure and incident AD risk. The pooled result should be interpreted with extreme caution (Figure 6).

### 3.9 Evaluation of publication bias

**Figure 7:**
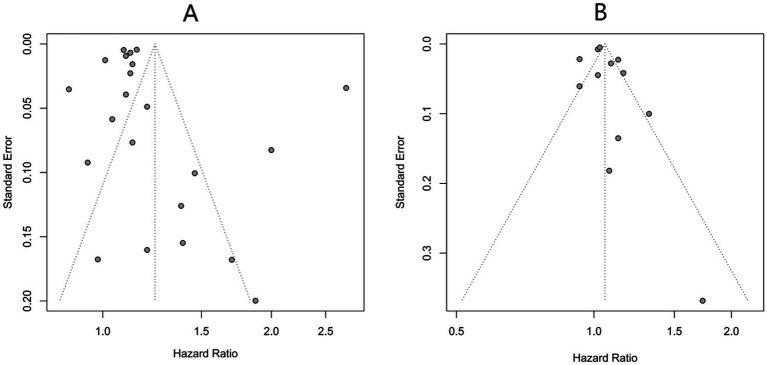
Funnel plots for publication bias of the association between exposure to PM_2.5_ **(A)** and NO_2_ **(B)** and incident AD risk.

Publication bias assessment results: For PM_2.5_ and NO_2_ (≥10 included studies), funnel plots showed symmetric distributions, suggesting low publication bias (Figure 7). For PM_10_, NOₓ, and O_3_ (<10 included studies), Egger’s test was used: PM_10_ (*p* = 0.159) and O_3_ (*p* = 0.395) had no significant publication bias; NOₓ (*p* = 0.047) showed potential bias, possibly due to underreporting of negative-result studies. The trim-and-fill method was applied to adjust for NOₓ’s potential publication bias. After imputing 1 missing negative-result study, the adjusted pooled HR for NOₓ was 1.02 (95% CI: 1.01–1.03, *p* < 0.001), which remained statistically significant.

## 4 Discussion

Based on individual data from 25 prospective or retrospective cohort studies, involving a total of over 170 million adults aged 50 years and above, this study estimated the dose–response relationships between long-term exposure to PM_2.5_, PM_10_, NO_2_, NOₓ, and O_3_ and the risk of incident AD simultaneously on a global scale for the first time. We found that for every 5 μg/m^3^ increase in PM_2.5_, the risk of AD incidence increased by 24% (HR = 1.24, 95% CI: 1.10–1.39). Compared with a 2025 study from the University of Cambridge, which was based on 26 million UK healthcare records (HR = 1.08 per 10 μg/m^3^) (34), our effect size was higher, which may be attributed to the following three factors: ① In our study, most of the “long-term exposure” was defined as a ≥ 5-year moving average, whereas the Cambridge study only used single-year baseline concentrations, which underestimated the cumulative dose; ② The baseline age of the population included in our study was ≥50 years, while the Cambridge database included a mix of people aged 40–45 years, and the younger dilution effect lowered the HR; ③ We used a random-effects model and retained studies with high heterogeneity, whereas the Cambridge study used a fixed-effects model, which may have excessively narrowed the confidence intervals.

Mechanistically, PM_2.5_’s neurotoxicity has been verified through three approaches: *in vivo*, *in vitro*, and imaging studies. A 2025 study of 602 autopsies found that PM2.5 exposure was significantly linked to the severity of Alzheimer’s disease neuropathological changes (ADNC) (35). Moreover, in a subgroup of 287 subjects with Clinical Dementia Rating Sum of Boxes (CDR-SB) data, this association was manifested as aggravated cognitive impairment (*β* = 0.48; 95% CI = 0.22–0.74). In 2025, Wei et al. (36) established a chronic exposure model by administering intranasal instillation of PM_2.5_ to APP/PS1 mice for 90 consecutive days. After 3 months, they found that PM_2.5_ exposure induced lysosomal dysfunction (e.g., altered membrane permeability and impaired degradation function) in the hippocampus and cortex of the mice, increased amyloid-beta (Aβ) plaque deposition and the Aβ42/Aβ40 ratio in the hippocampus and cortex, and simultaneously elevated the phosphorylation level of tau protein at Thr231—accelerating the pathological progression of AD. In terms of human brain imaging, the 2025 Seoul-Incheon Brain Imaging Study (*n* = 542, aged ≥ 65 years) revealed that for every 1 μg/m^3^ increase in indoor PM_2.5_, the bilateral hippocampal volume decreased by 55.4 mm3, and this association was independent of cerebrovascular risk factors. The authors also pointed out that hippocampal volume reduction is a key early imaging biomarker for AD, suggesting that air pollution-related cognitive impairment may be mediated by hippocampal atrophy (37).

The effects of NO_2_ and NO_x_ also warrant attention. We found that for every 10 μg/m^3^ increase in NO_2_, the risk of AD increased by 6% (HR = 1.06), with a stronger effect in the UK (HR = 1.13) and a significant difference from other European regions and Asia (*p* < 0.0001). This regional specificity is strongly supported by London’s traffic pollutant characteristics identified in a large-scale population study: ① Road traffic is the core source of key pollutants, with clear subdivision of PM_2.5_ sources into traffic exhaust and non-exhaust emissions (e.g., brake/tyre wear and resuspension), confirming traffic’s exclusive contribution to local air pollution (38). ② During 2006–2010, the average exposure to traffic-related NO2 in London reached 41 μg/m^3^, with NO_x_ as high as 73 μg/m^3^, while PM_2.5_ and PM_10_ averaged 14 μg/m^3^ and 23 μg/m^3^, respectively, representing sustained high-concentration exposure across the 2,317 km^2^ study area (39). A continental-scale attributable mortality study covering the entire continent in 2020 estimated that for every 10 μg/m3 increase in road transport-related NO_2_, the number of annual attributable premature deaths increased by approximately 9,300 cases (range: 5,500–14,000), which is equivalent to an independent contribution of NO_2_ to the increase in deaths of ≈ 1.5% (95% CI = 0.9–2.2%). This further supports the “high traffic source-high toxicity” hypothesis (40). In a 2-year follow-up of 9–10-year-old American children (ABCD Cohort, *n* = 9,497), every 10 μg/m^3^ increase in NO_2_ significantly reduced inter-network and cortico-network functional connectivity (*β* = −0.028, *p* < 0.001). This suggests that NO_2_ exposure during childhood can disrupt the maturation trajectory of brain networks, providing the first longitudinal evidence for the neurodevelopmental toxicity of NO_2_ (41). In the NO_x_ analysis, we found that the HR of the subgroup with >12 years of follow-up was 1.29, much higher than that of the subgroup with <12 years of follow-up (1.05). This further supports the notion that “cumulative dose” rather than “transient concentration” determines the risk of AD. This result is consistent with a 2024 British aging cohort study (*n* = 192,300), where cumulative NO_x_ exposure over the follow-up years increased the risk of dementia by 1.16 (1.10–1.21) (HR = 1.32). Additionally, 6.6% of the association between NOₓ and dementia was mediated by long-term metabolic disturbances, revealing for the first time that the cumulative neurotoxicity of NO_x_ can be exerted through lipid metabolism pathways (42). Notably, Egger’s test for NOₓ gave a *p*-value of 0.047, suggesting potential publication bias. This may result from underreporting of studies with negative results. We applied the trim-and-fill method and incorporated 1 imputed study. The adjusted pooled HR decreased to 1.02 (95% CI = 1.01–1.03) but remained statistically significant. This indicates that while potential publication bias cannot be excluded, the association between long-term NOₓ exposure and AD risk is still supported by the available data and not fully negated by the observed bias.

Surprisingly, a null result was observed for O_3_. After pooling data from 4 studies, we found that for every 60 μg/m^3^ increase in O_3_, the HR for AD risk was 1.04 (95% CI = 0.79–1.36), with extremely high heterogeneity (*I*^2^ = 99.8%). In-depth analysis showed discrepancies between two key studies: Jung et al. (Taiwan) (11) reported an HR of 3.12, while Carey et al. (UK) (13) reported an HR of 0.78. These differences stem from inherent variations in exposure assessment, co-exposure control, and definitions of study population and outcomes. Jung et al. (11) focused on individuals aged 65 years and above in Taiwan. For O_3_ exposure assessment, PM_2.5_ concentrations before 2006 had to be estimated using the mean ratio of PM_10_ to PM_2.5_ (0.57), which may have led to exposure misclassification. Additionally, O_3_ concentrations in Taiwan are generally high due to subtropical photochemical pollution, and the study did not clearly and fully isolate the synergistic effect of “O_3_ + PM_2.5_” mixed pollution. In contrast, Carey et al. (13) was based on a population aged 50–79 years in London. It used the KCLurban dispersion model with a 20 × 20 m resolution, combined with residential postcodes, to accurately assess O_3_ exposure. O_3_ concentrations in the UK are generally low due to the temperate maritime climate; moreover, in the multi-pollutant model, the study strictly adjusted for co-exposure pollutants such as NO_2_ and PM_2.5_, as well as confounding factors including area deprivation index and underlying diseases. These differences ultimately resulted in the divergence of effect sizes between the two studies. Animal experiments have shown that O_3_ exposure alone only induces mild neurodegenerative changes in mice. However, simultaneous exposure to O_3_ and PM_2.5_ not only leads to a synergistic enhancement of neuroinflammation but also is accompanied by a significant aggravation of blood–brain barrier damage and memory impairment. Furthermore, omics studies have confirmed that mitochondrial complex dysfunction in glial cells drives this synergistic effect (43). Therefore, the null result for O_3_ does not indicate the absence of neurotoxicity; instead, it emphasizes that the effect of O_3_ should be re-evaluated within the framework of mixed pollution, rather than relying on a single-pollutant model.

We observed high heterogeneity in this study. We conducted meta-regression (with exposure assessment method, follow-up duration, study region, and sample size as moderators) and further subgroup analysis stratified by exposure assessment method (the core heterogeneity source identified). Despite these efforts, the *I*^2^ value for PM_2.5_ remained above 80%—a level similar to the 80% heterogeneity reported in a meta-analysis on childhood asthma (44). We speculate that the core source lies in the fact that PM chemical composition was not captured. A review study has confirmed that differences in the contents of heavy metals and polycyclic aromatic hydrocarbons (PAHs) in PM_2.5_ can lead to differences in neurotoxicity by up to 3-fold (45). However, the studies included in this research did not report information such as black carbon or heavy metals, making it impossible to perform component-specific stratification. Second, there were differences in exposure assessment accuracy: most studies relied on regional models, which may underestimate the actual exposure level compared with individual biomarker monitoring. These unmeasured variables constitute residual heterogeneity, and future studies are required to collect more data to address this issue.

Based on the PM_2.5_ HR of 1.24 and the trend of no threshold effect, we recommend that the World Health Organization (WHO) tighten its annual guideline concentration from 5 μg/m^3^ to 3–4 μg/m^3^. Currently, there is substantial evidence showing that HEPA-filtered air purifiers can reduce indoor PM_2.5_ concentrations and improve subclinical health indicators (46). An existing systematic review, which included 16 studies [8 focusing on low-emission zones (LEZ) and 8 on congestion charging zones (CCZ)], revealed the following (47): LEZs have a positive impact on health outcomes related to air pollution—among the 6 studies evaluating cardiovascular diseases, 5 observed a reduction in the risk of certain subtypes of these diseases; for CCZs, taking London as an example, 6 out of 7 studies reported a decrease in road traffic injuries (RTIs). These pieces of evidence indicate that traffic emission reduction measures such as LEZs can effectively mitigate health risks associated with air pollution, further supporting the concept of “emission reduction equals prevention.” Such measures can be adopted as a primary prevention strategy for neurodegenerative diseases like AD.

At the same time, the limitations of this study should also be viewed objectively. First, due to data limitations of the included studies, it was not possible to conduct a dose–response relationship analysis. This made it impossible to identify the specific thresholds for the association between exposure to various pollutants and AD incidence risk, and difficult to further quantify the risk differences under different exposure levels. Second, the study lacks data on the chemical composition of pollutants, so it cannot distinguish the differences in AD-inducing activity among different components in PM_2.5_ [e.g., heavy metals, polycyclic aromatic hydrocarbons (PAHs)], making it hard to accurately identify the core pathogenic components. Furthermore, the conclusion that there is no clear association between O_3_ and AD incidence risk may also be limited by the small sample size—only 4 studies were included. The stability of this result still requires further verification by more high-quality, large-sample studies.

## 5 Conclusion

In summary, this study, conducted in a global population of 170 million individuals, confirmed that long-term exposure to PM_2.5_, NO_2_, and NO_x_ is associated with a robust positive correlation with the risk of AD incidence, with effect sizes higher than those reported in previous studies. Over the next 5 years, if combined progress and breakthroughs can be achieved in three key areas—research on pollutant components, analysis of genetic influences, and exploration of intervention measures—air pollution is expected to become the first environmental risk factor for AD that can be significantly adjusted and improved on a large scale. This will provide a practical primary prevention strategy for AD in aging societies worldwide.

## Data Availability

The original contributions presented in the study are included in the article/supplementary material, further inquiries can be directed to the corresponding author.

## References

[1] Lancet Public Health. Reinvigorating the public health response to dementia. Lancet Public Health. (2021) 6:e696. doi: 10.1016/S2468-2667(21)00215-2 34563278 PMC8516159

[2] Zhang H Wei W Zhao M Ma L Jiang X Pei H . Interaction between aβ and tau in the pathogenesis of Alzheimer's disease. Int J Biol Sci. (2021) 17:2181–92. doi: 10.7150/ijbs.57078, 34239348 PMC8241728

[3] Zheng Q Wang X. Alzheimer's disease: insights into pathology, molecular mechanisms, and therapy. Protein Cell. (2025) 16:83–120. doi: 10.1093/procel/pwae026, 38733347 PMC11786724

[4] Jones A Ali MU Mayhew A Aryal K Correia RH Dash D . Environmental risk factors for all-cause dementia, Alzheimer's disease dementia, vascular dementia, and mild cognitive impairment: an umbrella review and meta-analysis. Environ Res. (2025) 270:121007. doi: 10.1016/j.envres.2025.121007 39889875

[5] Zeng HX Qin SJ Andersson J Li SP Zeng QG Li JH . The emerging roles of particulate matter-changed non-coding RNAs in the pathogenesis of Alzheimer's disease: a comprehensive in silico analysis and review. Environ Pollut. (2025) 366:125440. doi: 10.1016/j.envpol.2024.125440, 39631655

[6] Wilker EH Osman M Weisskopf MG. Ambient air pollution and clinical dementia: systematic review and meta-analysis. BMJ. (2023) 381:e071620. doi: 10.1136/bmj-2022-071620, 37019461 PMC10498344

[7] Liu C Meng L Gao Y Chen J Zhu M Xiong M . PM2.5 triggers tau aggregation in a mouse model of tauopathy. JCI Insight. (2024) 9. doi: 10.1172/jci.insight.176703 PMC11383351 39133647

[8] Gialluisi A Costanzo S Veronesi G Cembalo A Tirozzi A Falciglia S . Prominent role of PM10 but not of circulating inflammation in the link between air pollution and the risk of neurodegenerative disorders. medRxiv. (2023) [Preprint]. doi: 10.1101/2023.05.17.23289154

[9] Soeterboek J Deckers K van Boxtel MPJ Backes WH Eussen S van Greevenbroek MMJ . Association of ambient air pollution with cognitive functioning and markers of structural brain damage: the Maastricht study. Environ Int. (2024) 192:109048. doi: 10.1016/j.envint.2024.109048 39383768

[10] Zhang Z Chen L Wang X Wang C Yang Y Li H . Associations of air pollution and genetic risk with incident dementia: a prospective cohort study. Am J Epidemiol. (2023) 192:182–94. doi: 10.1093/aje/kwac188, 36269005

[11] Jung CR Lin YT Hwang BF. Ozone, particulate matter, and newly diagnosed Alzheimer's disease: a population-based cohort study in Taiwan. J Alzheimer's Dis. (2015) 44:573–84. doi: 10.3233/JAD-140855, 25310992

[12] Kioumourtzoglou MA Schwartz JD Weisskopf MG Melly SJ Wang Y Dominici F . Long-term PM2.5 exposure and neurological hospital admissions in the northeastern United States. Environ Health Perspect. (2016) 124:23–9. doi: 10.1289/ehp.1408973, 25978701 PMC4710596

[13] Carey IM Anderson HR Atkinson RW Beevers SD Cook DG Strachan DP . Are noise and air pollution related to the incidence of dementia? A cohort study in London, England. BMJ Open. (2018) 8:e022404. doi: 10.1136/bmjopen-2018-022404, 30206085 PMC6144407

[14] Oudin A Andersson J Sundström A Nordin Adolfsson A Oudin Åström D Adolfsson R . Traffic-related air pollution as a risk factor for dementia: no clear modifying effects of APOEɛ4 in the Betula cohort. J Alzheimer's Dis. (2019) 71:733–40. doi: 10.3233/JAD-181037, 31450491

[15] Mortamais M Gutierrez LA de Hoogh K Chen J Vienneau D Carrière I . Long-term exposure to ambient air pollution and risk of dementia: results of the prospective Three-City study. Environ Int. (2021) 148:106376. doi: 10.1016/j.envint.2020.106376, 33484961

[16] Ran J Schooling CM Han L Sun S Zhao S Zhang X . Long-term exposure to fine particulate matter and dementia incidence: a cohort study in Hong Kong. Environ Pollut. (2021) 271:116303. doi: 10.1016/j.envpol.2020.116303, 33370610

[17] Shi L Steenland K Li H Liu P Zhang Y Lyles RH . A national cohort study (2000–2018) of long-term air pollution exposure and incident dementia in older adults in the United States. Nat Commun. (2021) 12:6754. doi: 10.1038/s41467-021-27049-2, 34799599 PMC8604909

[18] Parra KL Alexander GE Raichlen DA Klimentidis YC Furlong MA. Exposure to air pollution and risk of incident dementia in the UK biobank. Environ Res. (2022) 209:112895. doi: 10.1016/j.envres.2022.112895, 35149105 PMC8976829

[19] Shi LH Zhu Q Wang YF Hao H Zhang HS Schwartz J . Incident dementia and long-term exposure to constituents of fine particle air pollution: a national cohort study in the United States. Proc Natl Acad Sci USA. (2022) 120:e2211282119. doi: 10.1073/pnas.2211282119 36574646 PMC9910468

[20] Trevenen ML Heyworth J Almeida OP Yeap BB Hankey GJ Golledge J . Ambient air pollution and risk of incident dementia in older men living in a region with relatively low concentrations of pollutants: the health in men study. Environ Res. (2022) 215:114349. doi: 10.1016/j.envres.2022.114349, 36116491

[21] Yang L Wan WJ Yu CY Xuan C Zheng PN Yan J. Associations between PM2.5 exposure and Alzheimer's disease prevalence among elderly in eastern China. Environ Health. (2022) 21:119. doi: 10.1186/s12940-022-00937-w 36447194 PMC9706836

[22] Younan D Wang XH Gruenewald T Gatz M Serre ML Vizuete W . Racial/ethnic disparities in Alzheimer's disease risk: role of exposure to ambient fine particles. J Gerontol A Biol Sci Med Sci. (2022) 77:977–85. doi: 10.1093/gerona/glab231, 34383042 PMC9071399

[23] Chen GC Nyarko Hukportie D Wan Z Li FR Wu XB. The association between exposure to air pollution and dementia incidence: the modifying effect of smoking. J Gerontol A Biol Sci Med Sci. (2023) 78:2309–17. doi: 10.1093/gerona/glac228, 36373950

[24] Shim JI Byun G Lee JTT. Long-term exposure to particulate matter and risk of Alzheimer's disease and vascular dementia in Korea: a national population-based cohort study. Environ Health. (2023) 22:35. doi: 10.1186/s12940-023-00986-9 37060077 PMC10105439

[25] Yuan S Huang X Zhang L Ling Y Tan S Peng M . Associations of air pollution with all-cause dementia, Alzheimer's disease, and vascular dementia: a prospective cohort study based on 437,932 participants from the UK biobank. Front Neurosci. (2023) 17:1216686. doi: 10.3389/fnins.2023.1216686, 37600021 PMC10436530

[26] Zhu Z Yang Z Yu L Xu L Wu Y Zhang X . Residential greenness, air pollution and incident neurodegenerative disease: a cohort study in China. Sci Total Environ. (2023) 878:163173. doi: 10.1016/j.scitotenv.2023.163173, 37003317

[27] Jutila OEI Mullin D Vieno M Tomlinson S Taylor A Corley J . Life-course exposure to air pollution and the risk of dementia in the Lothian birth cohort 1936. Environ Epidemiol. (2024) 9:e355. doi: 10.1097/ee9.0000000000000355, 39669703 PMC11634326

[28] Peters S Bouma F Hoek G Janssen N Vermeulen R. Air pollution exposure and mortality from neurodegenerative diseases in the Netherlands: a population-based cohort study. Environ Res. (2024) 259:119552. doi: 10.1016/j.envres.2024.119552, 38964584

[29] Zhang Y Fu Y Guan X Wang C Fu M Xiao Y . Associations of ambient air pollution exposure and lifestyle factors with incident dementia in the elderly: a prospective study in the UK biobank. Environ Int. (2024) 190:108870. doi: 10.1016/j.envint.2024.108870, 38972114

[30] Qin MM Khoshnevis N Dominici F Braun D Zanobetti A Mork D. Comparing traditional and causal inference methodologies for evaluating impacts of long-term air pollution exposure on hospitalization with Alzheimer disease and related dementias. Am J Epidemiol. (2025) 194:64–72. doi: 10.1093/aje/kwae133, 38907309 PMC11735961

[31] Zhang H Wang Y Li H Zhu Q Ma T Liu Y . The role of the components of PM(2.5) in the incidence of Alzheimer's disease and related disorders. Environ Int. (2025) 200:109539. doi: 10.1016/j.envint.2025.109539, 40412353 PMC12185172

[32] Zheng L Su B Cui FP Li D Ma Y Xing M . Long-term exposure to PM(2.5) constituents, genetic susceptibility, and incident dementia: a prospective cohort study among 0.2 million older adults. Environ Sci Technol. (2025) 59:4493–504. doi: 10.1021/acs.est.4c08188, 39998422

[33] Zhu Q Deng YL Liu Y Steenland K. Associations between ultrafine particles and incident dementia in older adults. Environ Sci Technol. (2025) 59:5443–51. doi: 10.1021/acs.est.4c10574, 40079183 PMC11948469

[34] Best Rogowski CB Bredell C Shi Y Tien-Smith A Szybka M Fung KW . Long-term air pollution exposure and incident dementia: a systematic review and meta-analysis. Lancet Planet Health. (2025) 9:101266. doi: 10.1016/S2542-5196(25)00118-4, 40716448

[35] Kim B Blam K Elser H Xie SX Van Deerlin VM Penning TM . Ambient air pollution and the severity of Alzheimer disease neuropathology. JAMA Neurol. (2025) 82:1153. doi: 10.1001/jamaneurol.2025.3316, 40920417 PMC12418217

[36] Wei M Li W Bao G Yang Z Li S Le W. PM2.5 exposure exacerbates Alzheimer's disease pathology through lysosomal dysfunction in APP/PS1 mice. Ecotoxicol Environ Saf. (2025) 303:118918. doi: 10.1016/j.ecoenv.2025.118918, 40865239

[37] An SM Kim HH. The association between indoor air pollutants and brain structure indicators using eTIV-adjusted and unadjusted models: a study in Seoul and Incheon. Brain Sci. (2025) 15:868. doi: 10.3390/brainsci15080868, 40867199 PMC12384879

[38] Tonne C Halonen JI Beevers SD Dajnak D Gulliver J Kelly FJ . Long-term traffic air and noise pollution in relation to mortality and hospital readmission among myocardial infarction survivors. Int J Hyg Environ Health. (2016) 219:72–8. doi: 10.1016/j.ijheh.2015.09.003, 26454658

[39] Smith RB Fecht D Gulliver J Beevers SD Dajnak D Blangiardo M . Impact of London's road traffic air and noise pollution on birth weight: retrospective population based cohort study. BMJ. (2017) 359:j5299. doi: 10.1136/bmj.j5299 29208602 PMC5712860

[40] Arter CA Buonocore JJ Isakov V Pandey G Arunachalam S. Air pollution benefits from reduced on-road activity due to COVID-19 in the United States. PNAS Nexus. (2024) 3:pgae017. doi: 10.1093/pnasnexus/pgae017, 38292536 PMC10825624

[41] Cotter DL Campbell CE Sukumaran K McConnell R Berhane K Schwartz J . Effects of ambient fine particulates, nitrogen dioxide, and ozone on maturation of functional brain networks across early adolescence. Environ Int. (2023) 177:108001. doi: 10.1016/j.envint.2023.108001, 37307604 PMC10353545

[42] Tian F Wang Y Huang Z Qian AM Wang C Tan L . Metabolomic profiling identifies signatures and biomarkers linking air pollution to dementia risk: a prospective cohort study. J Hazard Mater. (2024) 480:136498. doi: 10.1016/j.jhazmat.2024.136498, 39547039

[43] Yang L Zhao S Wu Q Zeng Y Zhang A Sun H . Ozone and PM(2.5) co-exposure induced neurodegenerative alterations in mice: implication of mitochondrial dysfunction in glial cells. Environ Int. (2025) 204:109802. doi: 10.1016/j.envint.2025.109802 40974833

[44] Keleb A Abeje ET Daba C Endawkie A Tsega Y Abere G . The odds of developing asthma and wheeze among children and adolescents exposed to particulate matter: a systematic review and meta-analysis. BMC Public Health. (2025) 25:1225. doi: 10.1186/s12889-025-22382-3, 40165124 PMC11959839

[45] Li B Ma Y Zhou Y Chai E. Research progress of different components of PM(2.5) and ischemic stroke. Sci Rep. (2023) 13:15965. doi: 10.1038/s41598-023-43119-5, 37749193 PMC10519985

[46] Allen RW Barn P. Individual- and household-level interventions to reduce air pollution exposures and health risks: a review of the recent literature. Curr Environ Health Rep. (2020) 7:424–40. doi: 10.1007/s40572-020-00296-z, 33241434 PMC7749091

[47] Chamberlain RC Fecht D Davies B Laverty AA. Health effects of low emission and congestion charging zones: a systematic review. Lancet Public Health. (2023) 8:e559–74. doi: 10.1016/S2468-2667(23)00120-2, 37393094

[48] Jack CR Jr Bennett DA Blennow K Carrillo MC Dunn B Haeberlein SB . NIA-AA research framework: toward a biological definition of Alzheimer's disease. Alzheimers Dement. (2018) 14:535–62. doi: 10.1016/j.jalz.2018.02.018, 29653606 PMC5958625

